# Induction and expression of GluA1 (GluR-A)-independent LTP in the hippocampus

**DOI:** 10.1111/j.1460-9568.2009.06677.x

**Published:** 2009-03

**Authors:** Carola Romberg, Joel Raffel, Lucy Martin, Rolf Sprengel, Peter H Seeburg, J Nicholas P Rawlins, David M Bannerman, Ole Paulsen

**Affiliations:** 1 Department of Physiology, Anatomy and Genetics, University of OxfordOxford OX1 3PT, UK; 2 Department of Experimental Psychology, University of OxfordOxford, UK; 3 Max-Planck Institute of Medical Research, Department of Molecular NeurobiologyHeidelberg, Germany

**Keywords:** glutamate receptor, mouse, synaptic plasticity

## Abstract

Long-term potentiation (LTP) at hippocampal CA3–CA1 synapses is thought to be mediated, at least in part, by an increase in the postsynaptic surface expression of α-amino-3-hydroxy-5-methyl-4-isoxazole proprionic acid (AMPA) receptors induced by *N*-methyl-d-aspartate (NMDA) receptor activation. While this process was originally attributed to the regulated synaptic insertion of GluA1 (GluR-A) subunit-containing AMPA receptors, recent evidence suggests that regulated synaptic trafficking of GluA2 subunits might also contribute to one or several phases of potentiation. However, it has so far been difficult to separate these two mechanisms experimentally. Here we used genetically modified mice lacking the GluA1 subunit (*Gria1*^−/−^ mice) to investigate GluA1-independent mechanisms of LTP at CA3–CA1 synapses in transverse hippocampal slices. An extracellular, paired theta-burst stimulation paradigm induced a robust GluA1-independent form of LTP lacking the early, rapidly decaying component characteristic of LTP in wild-type mice. This GluA1-independent form of LTP was attenuated by inhibitors of neuronal nitric oxide synthase and protein kinase C (PKC), two enzymes known to regulate GluA2 surface expression. Furthermore, the induction of GluA1-independent potentiation required the activation of GluN2B (NR2B) subunit-containing NMDA receptors. Our findings support and extend the evidence that LTP at hippocampal CA3–CA1 synapses comprises a rapidly decaying, GluA1-dependent component and a more sustained, GluA1-independent component, induced and expressed via a separate mechanism involving GluN2B-containing NMDA receptors, neuronal nitric oxide synthase and PKC.

## Introduction

An eagerly discussed question in neuroscience is whether long-term potentiation (LTP; Bliss & Lømo, 1973;Bliss & Collingridge, 1993) engages the same neural mechanisms that are responsible for learning and memory (Martin *et al.*, 2000; Martin & Morris, 2002). However, the complex mechanisms underlying LTP are still not completely understood. Conventional hippocampal CA3–CA1 LTP (Bliss & Collingridge, 1993) is believed to be expressed, at least in part, by an increase in synaptic α-amino-3-hydroxy-5-methyl-4-isoxazole proprionic acid (AMPA) receptors (AMPAR). Most AMPAR in hippocampal neurons are heterodimers composed of either GluA1/GluA2 or GluA2/GluA3 subunit combinations (Wenthold *et al.*, 1996), using the new subunit nomenclature recommended by the International Union of Basic and Clinical Pharmacology (IUPHAR). Accordingly, AMPAR subunits earlier known as either GluR-A, B, C and D, or GluR1, 2, 3 and 4, are renamed GluA1, GluA2, GluA3 and GluA4, and the corresponding mouse genes are *Gria1*, *Gria2*, *Gria3* and *Gria4* (Collingridge *et al.*, 2009). In an influential study, Shi *et al.* (2001) suggested that LTP is mediated by an activity-regulated increase in synaptic GluA1-containing AMPAR. Consistent with this suggestion, Zamanillo *et al.* (1999) reported that LTP is absent in *Gria1*^−/−^ mice and concluded that the GluA1 subunit is an absolute requirement for tetanus-induced CA3–CA1 LTP.

In contrast to GluA1/GluA2 AMPAR, GluA2/GluA3 AMPAR are thought to constitutively cycle in and out of the synapse (Shi *et al.*, 2001). However, increasing evidence suggests that the constitutive recycling of GluA2-containing AMPAR might not be that passive after all: Daw *et al.* (2000) found that GluA2 surface expression is regulated by a protein kinase C (PKC)-dependent mechanism, and regulated insertion of GluA2 might be critical for the longer-term expression and maintenance of LTP in the hippocampus (Gardner *et al.*, 2005; Sossa *et al.*, 2007; Yao *et al.*, 2008). Furthermore, activity-driven GluA2 insertion mediates a form of LTP in the cerebellum (Kakegawa & Yuzaki, 2005), where the increase of GluA2-containing AMPAR is regulated by nitric oxide (Huang *et al.*, 2005), produced by neuronal nitric oxide synthase (nNOS) upon *N*-methyl-d-aspartate (NMDA) receptor (NMDAR) activation (Bredt & Snyder, 1989; Garthwaite *et al.*, 1989; Brenman & Bredt, 1997). A similar, nitric oxide-regulated surface expression of GluA2 has recently been reported in cultured hippocampal neurons (Sossa *et al.*, 2006, 2007). Moreover, Hoffman *et al.* (2002) provided initial evidence that hippocampal LTP can be expressed without the GluA1 subunit: a theta-burst induction paradigm revealed a gradually developing form of LTP in *Gria1*^−/−^ mice. However, the precise mechanisms underlying this GluA1-independent form of hippocampal LTP remained unclear.

We used extracellular field recordings from hippocampal slices of *Gria1*^−/−^ mice (Zamanillo *et al.*, 1999) to study the cellular mechanisms of GluA1-independent CA3–CA1 LTP. Extracellular, paired theta-burst stimulation induced robust LTP in *Gria1*^−/−^ mice without the early, rapidly decaying component characteristic of LTP in wild-type mice. Induction of GluA1-independent LTP specifically required the GluN2B (NR2B) NMDAR subunit. Moreover, GluA1-independent LTP was abolished by inhibitors of nNOS and PKC, two enzymes that participate in the activity-regulated insertion of GluA2-containing AMPAR (Daw *et al.*, 2000; Perez *et al.*, 2001; Huang *et al.*, 2005; Sossa *et al.*, 2006, 2007). Our data extend the evidence that CA3–CA1 LTP comprises a rapidly decaying, GluA1-dependent phase and a more sustained, GluA1-independent phase induced and expressed through a separate, GluN2B-, nNOS- and PKC-dependent mechanism.

## Materials and methods

### Mice

All animal care and procedures described below were in compliance with the UK Home Office Project and Personal licenses held by the authors in accordance with Home Office regulations under the Animals (Scientific Procedures) Act of 1986.

*Gria1*^−/−^ mice were created on a hybrid C57BL/6/129sv background (Zamanillo *et al.*, 1999). Several *Gria1*^−/−^ breeding pairs (*>*F4) were transported to the UK and backcrossed to C57BL/6. For experimental cohorts of wild-type and *Gria1*^−/−^ mice, *Gria1*^+/−^ mice were intercrossed on-site in the departmental animal holding facility. Mice were housed in groups of two–six with *ad libitum* access to food and water on a 12 : 12 h dark : light cycle at a temperature of ∼22 *°*C and a humidity of 60–70%. For the following experiments, only male mice were used to avoid variations due to physiological differences between sexes.

### Slice preparation

Horizontal hippocampal slices (350 μm) were prepared from adult male *Gria1*^−/−^ mice of at least 6 months of age and their male wild-type littermates, after decapitation following isoflurane-induced anaesthesia (Isocare, Animalcare, York, UK) in a desiccator (0.5 mL isoflurane, until breathing slowed down to approximately one breath per second, and stimulation of the limb withdrawal reflex no longer elicited a response). Slices were stored at 34 °C in an interface recording chamber, and superfused (2 mL/min) with artificial cerebrospinal fluid (aCSF) of the following composition (in mm): NaCl, 126; KCl, 3; NaH_2_PO_4_, 1.25; MgSO_4_, 2; CaCl_2_, 2; NaHCO_3_, 26; glucose, 10; bubbled with carbogen gas (95% O_2_–5% CO_2_) to pH 7.2–7.4.

### Electrophysiological protocols

**Fig. 1:**
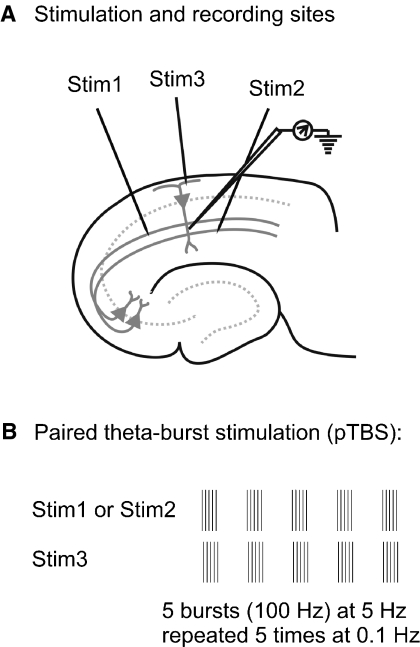
Position of electrodes and LTP induction paradigm. (A) Diagram of a transverse hippocampal slice showing the position of stimulation and recording electrodes. (B) Schematic illustration of the paired theta-burst stimulation (pTBS) LTP induction paradigm.

Extracellular field recordings from CA1 were made with an Axoclamp-2A amplifier (Axon Instruments, Union City, CA, USA) in bridge mode. Glass recording electrodes were pulled from standard wall borosilicate tubing (4–6 MΩ) and filled with aCSF. Recording and stimulation electrodes were positioned in CA1 with the aid of a stereoscope (Fig. 1A).

Blind patch-clamp recordings (Castañeda-Castellanos *et al.*, 2006) were made from CA1 pyramidal neurons with a Multiclamp 700B amplifier (Axon Instruments) in current-clamp mode. Patch pipettes (6–8 MΩ) were pulled from standard wall borosilicate tubing and filled with a solution containing (in mm): potassium gluconate, 110; HEPES, 40; NaCl, 4; ATP-Mg, 4; biocytin; GTP, 0.3; pH 7.2–7.3. The patch pipette was positioned just above the surface of the pyramidal cell layer of CA1 under visual guidance and then slowly lowered while constantly monitoring the current during voltage-clamp, in response to a test voltage step. Once whole-cell configuration was achieved, cells were recorded in current-clamp. Pyramidal neurons were identified according to their firing pattern and by biocytin labelling after the experiment, following standard procedures as described by Oren *et al.* (2006).

**Fig. 2:**
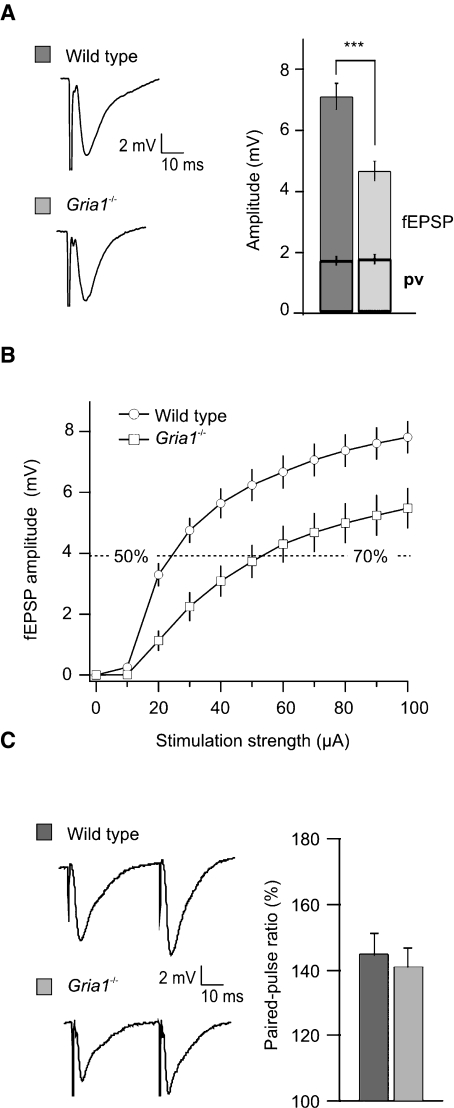
Synaptic transmission in *Gria1*^−/−^ mice. (A) Representative CA1 field excitatory postsynaptic potentials (EPSPs) in slices from a wild-type mouse (top left) and a *Gria1*^−/−^ mouse (bottom left), and mean presynaptic fibre volley (pv) and fEPSP amplitudes elicited by a single rectangular current pulse (50 μs at 70 μA) in the stratum radiatum among the Schaffer collaterals (wild-type, *n*=13; *Gria1*^−/−^, *n*=21), ***P<0.001 (anova). (B) Stimulation strength–response curves for wild-type (*n*=24) and *Gria1*^−/−^ mice (*n*=26). To compensate for reduced synaptic transmission in *Gria1*^−/−^ mice, the baseline stimulation strength for wild-type and *Gria1*^−/−^ mice in subsequent experiments was set to elicit an fEPSP of 50% and 70% of the maximal fEPSP amplitude, respectively. (C) Example traces of paired-pulse responses (left, 40-ms stimulus interval) and mean PPR in wild-type (*n*=6) and *Gria1*^−/−^ mice (*n*=5). Data are presented as mean ± SEM.

Synaptic efficacy was monitored in two independent afferent Schaffer collateral pathways stimulated alternately, each at 0.1 Hz (50 μs, 10–100 μA), with monopolar tungsten electrodes placed either side of the recording electrode (Fig. 1A). For field recordings, a stimulus–response curve [10–100 μA stimulation strength, mean of five field excitatory postsynaptic potentials (fEPSPs) at each stimulation strength] was established and the stimulation strength subsequently set to elicit an fEPSP of half-maximal amplitude in wild-type mice and the corresponding amplitude in *Gria1*^−/−^ mice (70% of the half-maximal amplitude; Fig. 2B). The basal stimulation strength during whole-cell recording from individual pyramidal neurons was adjusted to evoke an EPSP peak amplitude of 5–10 mV in both genotypes. fEPSP or EPSP rising slopes (middle third of the rising phase, before the action potential threshold) were monitored for a baseline period of 20 min. If synaptic transmission was stable (< 20% change in fEPSP or EPSP slopes over 20 min), one of the two Schaffer collateral pathways was stimulated with either a tetanic (100 Hz for 1 s) or paired theta-burst LTP induction paradigm (Fig. 1B). The other pathway served as a control and was not stimulated during the induction protocol. For paired theta-burst stimulation, the intensity of the stimulus delivered through the third electrode in the alveus was adjusted to a level just sufficient to elicit an antidromic population spike at the recording site.

In order to measure paired-pulse ratio (PPR), two 50-μs pulses with an inter-pulse interval of 40 ms were given at a stimulation strength eliciting half-maximal fEPSP amplitude in response to the first stimulus. The protocol was repeated five times at 0.1 Hz.

### Pharmacology

Drugs were applied to the superfusate for at least 30 min (at least 1 h for intracellularly acting drugs) before the onset of baseline recording. To block NMDAR irrespective of GluN2 subunit composition, 30 μm 3-(R-2-carboxypiperazin-4-yl)-propyl-1-phosphonic acid (CPP; Tocris Biosciences, Bristol, UK) was applied to the superfusate. GluN2B-containing NMDAR were targeted by the ifenprodil-derivative (*αR,βS*)-α-(5-hydroxyphenyl)-β-methyl-4-(phenylmethyl)-1-piperidinepropanol (Ro 25-6981; Tocris; Fischer *et al.*, 1997), whereas (*R*)-[(*S*)-1-(4-bromo-phenyl)-ethylamino]-(2,3-dioxo-1,2,3,4-tetrahydroquinoxalin-5-yl)-methyl-phosphonic acid (NVP-AAM077; kindly provided by Dr Y. Auberson, Novartis AG, Switzerland; Auberson *et al.*, 2002) was used to inhibit GluN2A-containing NMDA receptors. To block Ca^2+^/calmodulin-dependent protein kinase (CaM kinase), 1 μm 4-[(2*S*)-2-[(5-isoquinolinylsulphonyl) methylamino]-3-oxo-3-(4-phenyl-1-piperazinyl)propyl] phenylisoquinolinesulphonic acid ester (KN-62; Tocris; Tokumitsu *et al.,* 1990) was added to the superfusate. PKC was inhibited using 2 μm 1,2-dimethoxy-12-methyl[1,3]benzodioxolo[5,6-c]phenanthridinium (chelerythrine; Tocris; Herbert *et al.*, 1990), which shows some selectivity for the atypical isoform protein kinase Mζ (PKMζ) over other PKC isoforms at this concentration (Ling *et al.*, 2002). To inhibit nNOS, either 100 μm of the membrane-permeable inhibitor (4S)-*N*-(4-amino-5[aminoethyl]aminopentyl)-*N*′-nitroguanidine (nNOS inhibitor I; Merck Biosciences, Nottingham, UK; Hah *et al.*, 2001) or 0.2 μm of vinyl-L*-N-*5-(1-imino-3-butenyl)-L-ornithine (L-VNIO; Alexis, Nottingham, UK), another NOS inhibitor with a high selectivity for nNOS (Hopper & Garthwaite, 2006), were applied to the aCSF. Unlike other drugs, nNOS inhibitors were added to the aCSF during dissection, slice preparation and throughout the recovery period in order to allow sufficient time for the drug to reach its intracellular target.

### Data analysis

Data acquisition and subsequent analysis were performed using Igor Pro (WaveMetrics, Lake Oswego, OR, USA), and statistical analysis was performed using spss software (SPSS, Chicago, IL, USA). Changes in synaptic efficacy were estimated by using the mean fEPSP or EPSP rising slopes of a specified 5-min period expressed as a percentage of the mean fEPSP or EPSP rising slope from the 5-min baseline period immediately before the LTP induction paradigm was delivered. PPR was expressed as the mean ratio of the rising slope of the second fEPSP to the rising slope of the first fEPSP in % (average of five paired pulses). All values are given as mean ± SEM, and statistical significance was assessed using the Student’s two-tailed *t*-test, anova and repeated-measures (RM) anova, followed by a *post hoc* analysis of simple main effects where applicable, using Sidak’s adjustments for multiple comparisons. Numbers (*n*) refer to the number of mice.

## Results

### Gria1^−/−^*mice show attenuated synaptic transmission at CA3–CA1 synapses*

To compare synaptic transmission at CA3–CA1 synapses in *Gria1*^−/−^ and wild-type mice, extracellular field recordings from the stratum radiatum of CA1 were made while stimulating the Schaffer collateral pathway with 50-μs current pulses at various amplitudes. In response to single 70-μA current pulses, the mean fEPSP in *Gria1*^−/−^ mice reached only 65% of the mean fEPSP amplitude in wild-type mice (Fig. 2A; one-way anova, *F*_1,32_ = 19.6, *P*<0.001). The smaller fEPSP amplitudes in *Gria1*^−/−^ mice were observed across a range of stimulation strengths from 10 to 100 μA (Fig. 2B; RM anova, main effect of genotype *F*_1,48_ = 11.7, *P <*0.005, genotype by stimulation strength interaction *F*_9,432_ = 5.8, *P <*0.05). The smaller fEPSP amplitude in *Gria1*^−/−^ mice could not be attributed to a reduction in fibre excitability, as the presynaptic fibre volley amplitudes were almost identical between the genotypes (Fig. 2A; one-way anova, *F <*1). Moreover, a comparison of PPRs in wild-type and *Gria1*^−/−^ mice showed no significant differences between genotypes and thus provided no evidence for an alteration of presynaptic signalling in *Gria1*^−/−^ mice (Fig. 2C; stimulus duration 50 μs, inter-stimulus interval 40 ms, at 70 μA, one-way anova of PPR, *F*<1; see also Zamanillo *et al.*, 1999). Hence, with equivalent presynaptic activation, the postsynaptic response was significantly smaller in *Gria1*^−/−^ mice. This result suggests that a higher stimulation strength is needed to produce a postsynaptic excitatory current in *Gria1*^−/−^ mice equivalent to that in wild-type mice. In the following experiments, the stimulation strength for monitoring basal synaptic transmission and for induction of LTP in *Gria1*^−/−^ mice was therefore set to 70% of the maximal amplitude, roughly corresponding to 50% of the maximal amplitude in wild-type mice (Fig. 2B).

### Extracellular paired theta-burst stimulation induces a robust GluA1-independent form of LTP

To induce CA3–CA1 LTP in *Gria1*^−/−^ mice, we adapted the intracellular theta-burst paradigm previously described by Hoffman *et al.* (2002) for extracellular field recordings. After stable baseline synaptic transmission for at least 20 min, one of the two Schaffer collateral input pathways was stimulated with a theta-burst paradigm that was paired, with a small temporal offset, with an identical theta-burst paradigm applied to the alveus of CA1 (pTBS; Fig. 1A and B). The rationale for this pairing paradigm was to elicit synaptic events coinciding with backpropagating action potentials in the postsynaptic neuronal population.

**Fig. 3:**
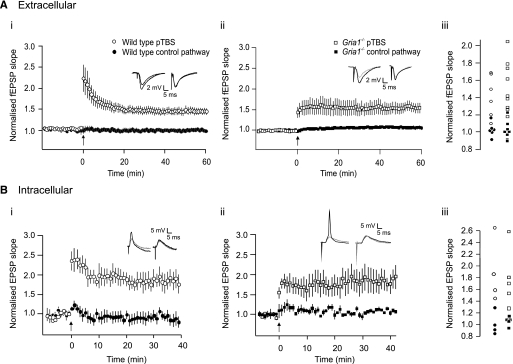
Hippocampal CA3–CA1 LTP in *Gria1*^−/−^ and wild-type mice. (A) Mean paired theta-burst stimulation (pTBS)-induced LTP of field excitatory postsynaptic potentials (fEPSPs) in (i) wild-type (*n*=6) and (ii) *Gria1*^−/−^ mice (*n*=8). The insets show example traces of the paired (left) and control pathways (right) before (grey) and 45 min after pTBS (black). (iii) Normalised fEPSP slope in each individual slice 45 min after pTBS in paired (open symbols) and control pathway (filled symbols). (B) The same paradigm induced input-specific LTP of EPSPs in individual CA1 pyramidal neurons of both (i) wild-type (*n*=4), and (ii) *Gria1*^−/−^ mice (*n*=5). The insets show example traces of the paired (left) and control pathways (right) before (grey) and 45 min after pTBS (black). (iii) Normalised EPSP slope in each individual cell 45 min after pTBS.

pTBS of the Schaffer collateral input/alveus induced significant LTP in wild-type mice (potentiation after 45 min: 150 ± 10%, Student’s *t*-test, *t*=4.0, *P*<0.05) as well as *Gria1*^−/−^ mice (154 ± 15%, *t*=3.6, *P*<0.01), whereas no fEPSP slope changes were observed in the unpaired control pathway (wild-type: 100 ± 5%, *t*<1; *Gria1*^−/−^: 105 ± 4%, *t*<1; Fig. 3A). However, the absence of an early, rapidly decaying component of potentiation readily distinguished LTP in *Gria1*^−/−^ mice from potentiation in their wild-type littermates. Despite this difference in the early potentiation, fEPSPs in both genotypes reached the same level of potentiation 45 min after induction. In order to compare the time course and amount of pTBS-induced potentiation in wild-type and *Gria1*^−/−^ mice, a RM anova was conducted on the normalised fEPSP slopes immediately (0–5 min), 15–20 min and 45–50 min after induction in both genotypes. The anova revealed no main effect of genotype (*F*<1), a main effect of time (*F*_2,24_ = 6.5, *P*<0.05), and a significant interaction of time and genotype (*F*_2,24_ = 8.9, *P*<0.01). A *post hoc* analysis of the simple main effects of genotype at each individual time point confirmed that the amount of potentiation in *Gria1*^−/−^ and wild-type mice differed immediately after pTBS (*F*_1,12_ = 12.7, *P*<0.001), but not 15 min or 45 min after induction (both *F*<1).

Although pTBS-induced LTP was pathway specific when observed with extracellular field recordings, the intracellular theta-burst stimulation originally used to induce GluA1-independent potentiation not only potentiated the EPSP in the paired pathway, but also caused a significant increase of EPSP amplitude in the control pathway (Hoffman *et al.*, 2002). Therefore, in order to test whether the GluA1-independent potentiation we observed with field recordings is a pathway-specific form of LTP also at the single-cell level, we recorded from individual pyramidal neurons in whole-cell mode and applied pTBS under experimental conditions identical to those described above for field recordings.

Firing patterns and *post hoc* biocytin labelling confirmed that the cells we recorded from were pyramidal neurons. As observed with field recordings, pTBS of the Schaffer collaterals/alveus induced similar amounts of LTP in CA1 pyramidal cells of *Gria1*^−/−^ mice (potentiation after 40 min: 181 ± 27%; Student’s *t*-test: *t*=3.9, *P*<0.05) and their wild-type littermates (potentiation after 40 min: 184 ± 27%; *t*=3.4, *P*<0.05; Fig. 3B). In both genotypes, LTP was observed only in the paired pathway, with no significant change in the control pathway in either wild-type (85 ± 17%, *t*<1) or *Gria1*^−/−^ mice (111 ± 5%, *t*<1). Intracellular, GluA1-independent LTP showed the same characteristic lack of an initial, rapidly decaying potentiation as LTP recorded in the field, and eventually reached the same level as intracellular LTP in wild-type mice. A RM anova comparing EPSP potentiation in the two genotypes immediately, and 40 min after induction, revealed no main effect of genotype or time (all *F*<2.5, *P*>0.10), but a significant genotype by time interaction (*F*_1,8_ = 25.8, *P*<0.005). A *post hoc* analysis of simple main effects showed a significant difference in the magnitude of potentiation between wild-type and *Gria1*^−/−^ mice immediately after pTBS (*F*_1,8_ = 6.1, *P*<0.05), but not 40 min after induction (*F*<1.4, *P*>0.20). Hence, both single-cell and field recordings show that the GluA1 subunit is not obligatory for input-specific LTP at Schaffer collateral–CA1 pyramidal cell synapses.

### A single weak tetanus is not sufficient to induce GluA1-independent LTP

**Fig. 4:**
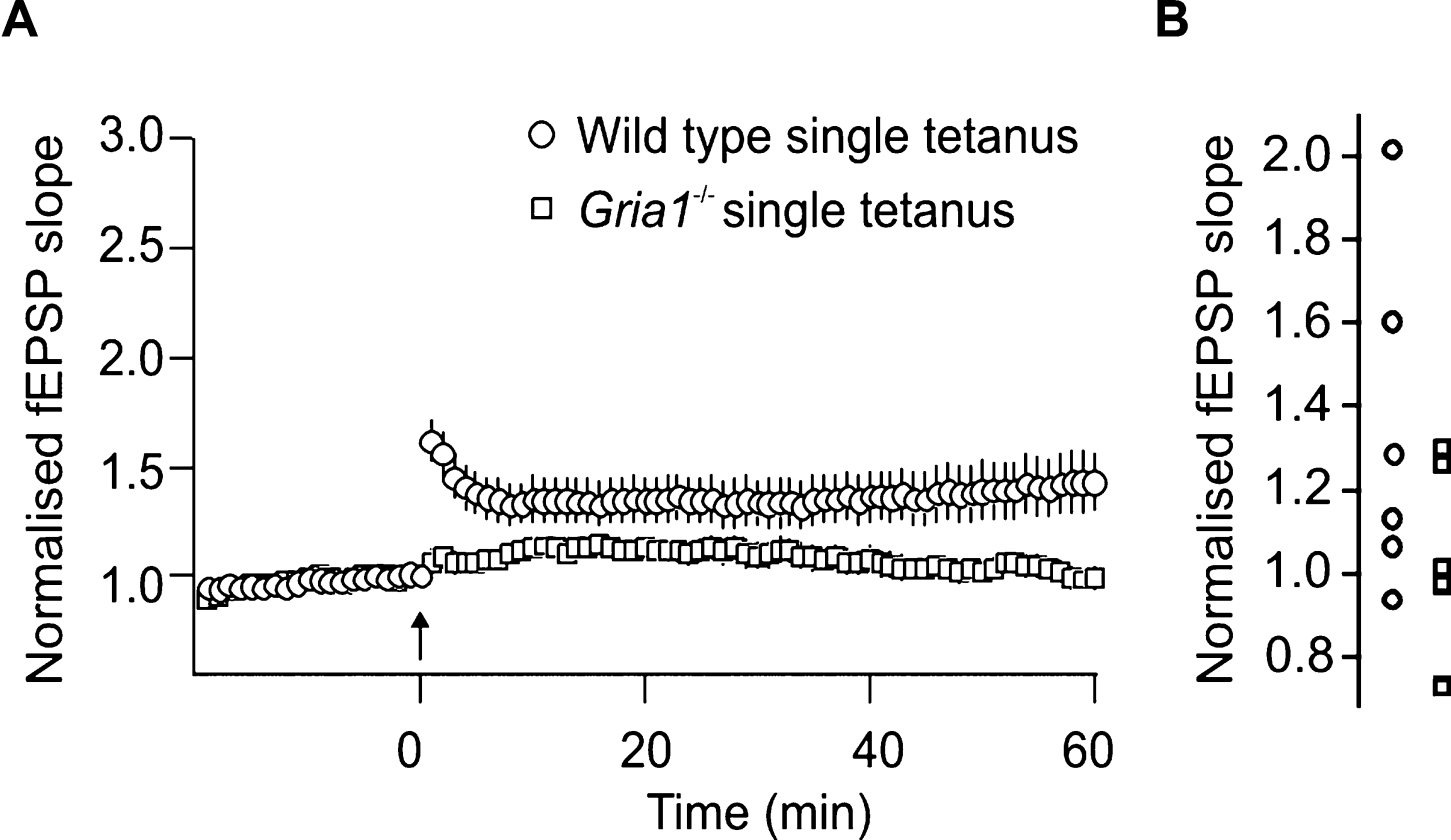
A single weak tetanus is not sufficient to induce LTP in *Gria1*^−/−^ mice. (A) Mean potentiation in wild-type (*n*=6) and *Gria1*^−/−^ mice (*n*=5) after a single tetanus to the Schaffer collateral pathway. Data are presented as mean ± SEM. (B) Normalised field excitatory postsynaptic potential (fEPSP) slope in each individual slice 45 min after the tetanus in wild-type (circles) and *Gria1*^−/−^ mice (squares).

Because previous studies reported a lack of LTP in adult *Gria1*^−/−^ mice (Zamanillo *et al.*, 1999; Jensen *et al.*, 2003), we also tested whether a single 100-Hz tetanus, as used by Zamanillo *et al.* (1999), would be sufficient to induce GluA1-independent LTP. Although this weaker induction paradigm led to small but significant LTP in wild-type mice (potentiation 45 min after induction: 135 ± 13%, *t*=2.65, *P*<0.05), no significant potentiation developed in *Gria1*^−/−^ mice (105 ± 10%, *t*<1; Fig. 4), which suggests that GluA1-independent LTP and GluA1-dependent potentiation have distinct induction properties.

### PPRs give no evidence for a presynaptic expression mechanism of GluA1-independent LTP

Both pre- and postsynaptic mechanisms can contribute to the expression of long-lasting changes in synaptic efficacy (see Malenka & Bear, 2004). Because the GluA1 subunit is important for several mechanisms implicated in postsynaptic LTP expression (Benke *et al.*, 1998; Derkach *et al.*, 1999; Hayashi *et al.*, 2000; Shi *et al.*, 2001; Piccini & Malinow, 2002; Boehm *et al.*, 2006; Oh *et al.*, 2006), it is possible that GluA1-independent LTP could be expressed presynaptically. A decrease in the PPR after LTP induction is commonly used to argue for the recruitment of presynaptic expression mechanism(s), because a presynaptic expression mechanism mediated by an increased probability of presynaptic transmitter release would leave less capacity for paired-pulse facilitation (McNaughton, 1982; Zucker & Regehr, 2002). However, neither GluA1-independent LTP nor LTP in wild-type mice was accompanied by a change in PPR (*Gria1*^−/−^: 140 ± 4% before pTBS, 141 ± 9% 45 min after pTBS; wild-type: 143 ± 4% before pTBS, 138 ± 5% 45 min after pTBS, RM anova all *F*<1). Thus, analysis of PPR provides no evidence for a presynaptic locus of expression of GluA1-independent LTP at hippocampal CA3–CA1 synapses.

### The induction of GluA1-independent LTP requires activation of GluN2B subunit-containing NMDAR

Because GluA1-independent LTP can be induced with a theta-burst pairing paradigm, but does not occur after a single weak tetanus, we investigated whether distinct NMDAR subunits are involved in the induction of GluA1-independent LTP mechanisms. At hippocampal synapses, NMDAR form GluN1/GluN2A, GluN1/GluN2B and GluN1/GluN2A/GluN2B heteromers (Monyer *et al.*, 1994; Cull-Candy *et al.*, 2001; Köhr, 2006), and recent evidence suggests that GluN2A and GluN2B subunits are linked to distinct intracellular signalling cascades (Liu *et al.*, 2004; Barria & Malinow, 2005; Kim *et al.*, 2005).

**Fig. 5:**
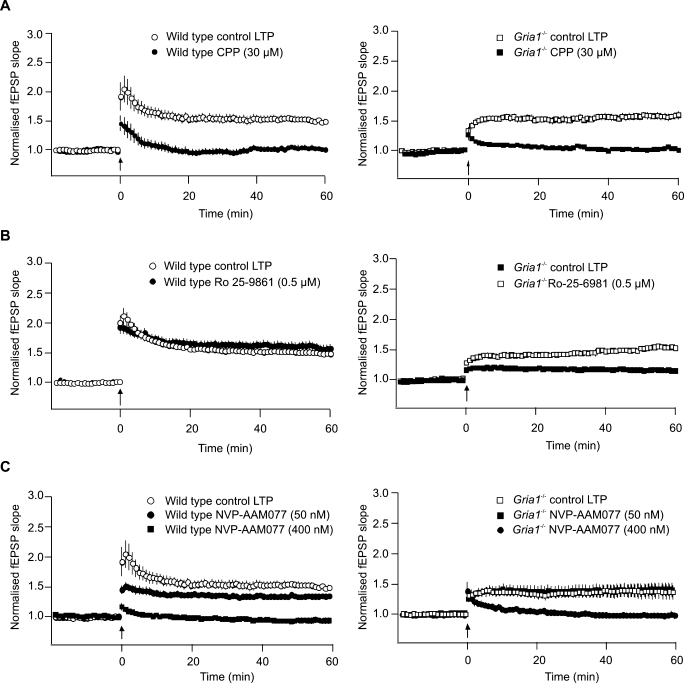
GluA1-independent long-term potentiation (LTP) requires activation of GluN2B-containing NMDA receptors. (A) The NMDAR antagonist 3-(R-2-carboxypiperazin-4-yl)-propyl-1-phosphonic acid (CPP) blocked pTBS-induced LTP in wild-type (*n*=7, control *n*=9) and *Gria1*^−/−^ mice (*n*=5, control *n*=7). (B) The GluN2B subunit-selective NMDAR antagonist (*αR,βS*)-α-(5-hydroxyphenyl)-β-methyl-4-(phenylmethyl)-1-piperidinepropanol (Ro 25-6981) had no effect on LTP in wild-type mice (*n*=9, control *n*=15), but attenuated LTP in *Gria1*^−/−^ mice (*n*=15, control *n*=11). (C) (*R*)-[(*S*)-1-(4-bromo-phenyl)-ethylamino]-(2,3-dioxo-1,2,3,4-tetrahydroquinoxalin-5-yl)-methyl-phosphonic acid (NVP-AAM077) (50 nm) had no significant effect on either GluA1-independent LTP (*n*=6, control *n*=6) or LTP in wild-type mice (*n*=12, control *n*=8). NVP-AAM077 (400 nm) attenuated both GluA1-independent LTP (*n*=9) and LTP in wild-type mice (*n*=7). Data are presented as mean ± SEM. fEPSP, field excitatory postsynaptic potential.

First, we tested whether pTBS-induced, GluA1-independent LTP and LTP in wild-type mice are similarly affected by the general NMDAR antagonist CPP (30 μm). As previously described by Hoffman *et al.* (2002) for the intracellular GluA1-independent LTP, the inhibition of NMDAR completely abolished the induction of GluA1-independent potentiation by an extracellular pTBS paradigm, as well as LTP in wild-type mice (Fig. 5A). A RM anova with drug as a between-subjects factor (CPP vs. control) and time as a within-subjects factor (0–5 min and 45–50 min after pTBS) for each genotype revealed a main effect of drug on LTP, both in *Gria1*^−/−^ mice (*F*_1,10_ = 49.2, *P*<0.005, drug by time interaction, *F*_1,10_ = 6.9, *P*<0.05), and in wild-type littermate controls (*F*_1,14_ = 21.4, *P*<0.001, main effect of time, *F*_1,14_ = 8.3, *P*<0.05, no further interactions, all *F*<1), which shows that NMDAR activation is necessary, both for the induction of the early, decaying phase of potentiation in wild-type mice and for GluA1-independent potentiation.

To target GluN2B-containing NMDAR, we used the GluN2B subunit-selective compound Ro 25-6981 (Fischer *et al.*, 1997). Superfusion of 0.5 μm Ro 25-6981 for at least 1 h prior to pTBS had no effect on the induction of LTP in wild-type mice, and in particular did not affect the early, rapidly decaying phase [Fig. 4B; RM anova with drug condition (Ro 25-6981 vs. control) as a between-subjects factor and time (0–5 min and 45–50 min after pTBS) as a within-subject factor, no effect of drug or interactions involving drug, all *F*<1]. In contrast, Ro 25-6981 strongly reduced GluA1-independent LTP (Fig. 5B; main effect of drug, *F*_1,24_ = 27.7, *P*<0.005, drug by time interaction, *F*_1,24_ = 7.9, *P <*0.001). Thus, induction of LTP requires the activation of GluN2B-containing NMDAR in *Gria1*^−/−^ mice, whereas the GluN2B subunit does not appear to be required when GluA1 is present.

In the next set of experiments, slices were treated with the GluN2A subunit-preferring NMDAR antagonist NVP-AAM077 (Auberson *et al.*, 2002). Because the selective action of NVP-AAM077 is concentration-dependent (Berberich *et al.*, 2005; Neyton & Paoletti, 2006), NVP-AAM077 was applied at two concentrations, 50 and 400 nm. In HEK293 cells expressing either GluN1/GluN2A- or GluN1/GluN2B-type rodent NMDAR, 50 nm NVP-AAM077 inhibits 77% of GluN2A-mediated NMDA receptor currents and 27% of GluN2B-mediated NMDA receptor currents, whereas 400 nm NVP-AAM077 blocks 97% of the GluN2A-mediated currents and 67% of GluN2B-mediated currents (Berberich *et al.*, 2005). When applied to the superfusate for at least 1 h prior to pTBS, 50 nm NVP-AAM077 had no overall effect on LTP in either wild-type or *Gria1*^−/−^ mice (all *F*<1, see overall RM anova results below). Although not strictly appropriate in the absence of a significant drug by time interaction (Howell, 1997), a separate *t*-test comparing the early, possibly GluA1-dependent component of potentiation in wild-type mice with and without NVP-AAM077 revealed a significant effect of drug (*t*=3.8, *P*<0.05). NVP-AAM077 (400 nm) in the superfusate entirely prevented the induction of LTP in both genotypes (Fig. 5C). A RM anova with drug (0, 50 and 400 nm NVP-AAM077) as a between-subjects factor and time (0–5 min and 45–50 min after pTBS) as a within-subject factor for each genotype revealed a main effect of drug on LTP in *Gria1*^−/−^ mice (*F*_2,18_ = 5.8, *P*<0.001, no further effects or interactions, all *F*<2, *P*>0.2) and wild-type mice (*F*_2,24_ = 12.6, *P*<0.001; main effect of time, *F*_1,24_ = 10.7, *P*<0.005; no interactions, all *F*<2). A *post hoc* comparison of the effect of 50 nm NVP-AAM077 returned no significant effect of 50 nm NVP-AAM077 (*Gria1*^−/−^: *F*<1, *P*>0.1; wild-type: *F*<1, *P*>0.1), and no significant drug by time interaction (*Gria1*^−/−^: *F*<1, *P*>0.2; wild-type: *F*<1, *P*>0.1) in either genotype. In contrast, a *post hoc* comparison of the effect of 400 nm NVP-AAM077 revealed a significant effect on LTP in wild-type (*F*_1,24_ = 12.6, *P*<0.001) and *Gria1*^−/−^ mice (*F*_1,18_ = 5.8, *P*<0.05).

Thus, only the early, possibly GluA1-dependent component of potentiation in wild-type mice was sensitive to NVP-AAM077 at low concentrations. The limited selectivity of NVP-AAM077 at higher concentrations prevented us from reaching a definitive conclusion about the contribution of GluN2A-containing NMDAR to induction of LTP in either genotype.

### GluA1-independent LTP requires CaM kinase

**Fig. 6:**
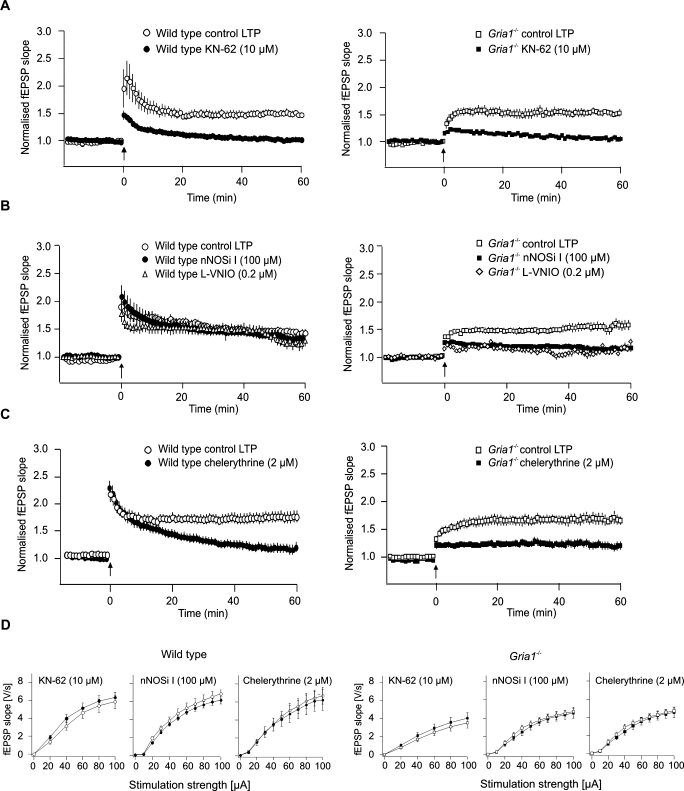
GluA1-independent long-term potentiation (LTP) requires CaM kinase, neuronal nitric oxide synthase (nNOS) and PKC. (A) GluA1-independent LTP (*n*=9, control *n*=4) and wild-type LTP (*n*=7, control *n*=6) are both attenuated by the CaM kinase inhibitor 4-[(2*S*)-2-[(5-isoquinolinylsulphonyl)methylamino]-3-oxo-3-(4-phenyl-1-piperazinyl)propyl] phenylisoquinolinesulphonic acid ester (KN-62). (B) nNOS inhibitor I and L-VNIO had no effect on the early potentiation in wild-type mice (left, nNOSi I: *n*=12; L-VNIO: *n*=5; control: *n*=5), whereas they strongly reduced GluA1-independent LTP (right, nNOSi I: *n*=14; L-VNIO: *n*=5; control: *n*=6). (C) Chelerythrine (2 μm) reduced GluA1-independent LTP (right, *n*=8, control *n*=9), and the later phase of potentiation in wild-type mice (left, *n*=13, control *n*=10). (D) Stimulus–response curves before and 3 h after application of CaM kinase, nNOS and PKC inhibitors in wild-type (left, KN-62: *n*=8; nNOSi I: *n*=9; chelerythrine: *n*=7) and *Gria1*^−/−^ mice (right, KN-62: *n*=12; nNOSi I: *n*=7; chelerythrine: *n*=9). fEPSP, field excitatory postsynaptic potential.

Downstream of NMDAR activation, a transient rise in intracellular Ca^2+^ levels is thought to be important for LTP induction (Lynch *et al.*, 1983; Collingridge *et al.*, 1992; Raymond & Redman, 2002). The enzyme CaM kinase II (CaMKII) acts as a Ca^2+^ sensor in the postsynaptic spine and is essential for LTP in wild-type mice (Malinow *et al.*, 1988, 1989; Silva *et al.*, 1992). GluA1, which can be phosphorylated by CaMKII on the S831 residue (Barria *et al.*, 1997; Lee *et al.*, 2000), is among the many target proteins of CaMKII that have been implicated in synaptic plasticity. We therefore asked whether a CaM kinase is still required for LTP when GluA1 is absent. The CaM kinase inhibitor KN-62 (10 μm) was applied for at least 2 h prior to pTBS, and had no effect on basal synaptic transmission in either genotype (Fig. 6D; RM anova*F*<1, *P*>0.3 for both genotypes). It completely abolished LTP in wild-type mice, and also attenuated LTP in their *Gria1*^−/−^ littermates (Fig. 6A). A RM anova with drug (KN-62 vs. control) as a between-subjects factor and time (0–5 min and 45–50 min after pTBS) as a within-subject factor returned a main effect of drug on LTP in *Gria1*^−/−^ (*F*_1,11_ = 29.7, *P*<0.001, no further effects or interactions, all *F*<1.9, *P*>0.2) and wild-type mice (*F*_1,11_ = 13.1, *P*<0.005, main effect of time, *F*_1,11_ = 8.9, *P*<0.05, no interactions, all *F*<1). Thus, CaM kinase appears to be necessary for the induction of LTP regardless of whether or not the GluA1 subunit is present.

### GluA1-independent LTP is mediated by a pathway involving nNOS and PKC

Although research on the expression mechanisms of LTP has focused mainly on the activity-driven synaptic insertion of GluA1 (Shi *et al.*, 2001), recent data from cultured cerebellar and hippocampal neurons have revealed a nitric oxide and *N*-ethylmaleimide-sensitive factor (NSF)-dependent pathway that leads to an increase in synaptic GluA2 subunits (Huang *et al.*, 2005; Sossa *et al.*, 2006, 2007). In order to assess whether this, or a similar, pathway mediates the GluA1-independent form of LTP in *Gria1*^−/−^ mice, extracellular field recordings were performed in the presence of a potent, membrane-permeable inhibitor of nNOS, 100 μm nNOS inhibitor I. This inhibitor had no effect on basal synaptic transmission in either genotype (Fig. 6D; RM anovas, *F*<1, *P*>0.2 for both genotypes) or on the amplitude of pTBS-induced LTP in wild-type mice [Fig. 6B; RM anova with drug (nNOS inhibitor I vs. control) as a between-subjects factor and time (0–5 min and 45–50 min after pTBS) as a within-subject factor: main effect of time, *F*_1,15_ = 7.0, *P*<0.05, no main effect of drug or interactions, all *F*<1]. In contrast, nNOS inhibitor I significantly reduced the magnitude of potentiation in *Gria1*^−/−^ mice [Fig. 6B; RM anova with drug (nNOS inhibitor I vs. control) as a between-subjects factor and time (0–5 min and 45–50 min after pTBS) as a within-subject factor, main effect of drug, *F*_1,18_ = 5.7, *P*<0.05, no further effects or interactions, all *F*<2, *P*>0.1]. Similar results were obtained with a second nNOS inhibitor, L-VNIO (0.2 μm), which had no significant effect on the first 60 min of potentiation in wild-type mice (Fig. 6B; RM anova, no main effect of drug or interactions, all *F*<2, *P*>0.1), but significantly reduced LTP in *Gria1*^−/−^ animals [Fig. 6B; RM anova with drug (L-VNIO vs. control) as a between-subjects factor and time (0–5 min and 45–50 min after pTBS) as a within-subject factor, main effect of drug, *F*_1,9_ = 3.9, *P*<0.05, no further effects or interactions, all *F*<1, *P*>0.1]. It therefore appears that nNOS plays an important role in the induction of GluA1-independent LTP, which supports the hypothesis that this form of LTP might be mediated by an insertion of GluA2-containing AMPAR via a regulated nNOS and possibly NSF-dependent pathway (Sossa *et al.*, 2006, 2007).

PKC is another protein involved in the synaptic trafficking of both GluA1 (Boehm *et al.*, 2006) and GluA2 AMPAR subunits (Daw *et al.*, 2000; Perez *et al.*, 2001), and particularly the PKMζ isoform plays an important role in the later phases of LTP in wild-type mice (Ling *et al.*, 2006; Yao *et al.*, 2008). We therefore tested whether PKC activity is required for the GluA1-independent form of LTP. Inhibition of PKC with chelerythrine (2 μm), an inhibitor that, at low concentrations, shows a preference for PKMζ over other PKC isoforms (Ling *et al.*, 2002), had no effect on basal synaptic transmission in either wild-type or *Gria1*^−/−^ mice (Fig. 6D; RM anovas, *F*<1, *P*>0.2 for both genotypes). In wild-type mice, chelerythrine had no effect on the magnitude of the initial potentiation immediately after pTBS, but significantly reduced the magnitude of LTP 45 min after induction (Fig. 6C). A RM anova with drug (chelerythrine vs. control) as a between-subjects factor and time (0–5 min and 45–50 min after induction) as a within-subject factor returned no effect of drug (*F*<1), but a significant drug by time interaction (*F*_1,21_ = 9.5, *P*<0.005). A *post hoc* simple main effects analysis of the effect of chelerythrine at each time point confirmed that there was no effect on the early, rapidly decaying potentiation in wild-type mice (*F*<1). In contrast, LTP 45 min after induction was significantly reduced (*F*_1,21_ = 7.1, *P*<0.05). In *Gria1*^−/−^ mice, chelerythrine (2 μm) strongly attenuated the overall level of potentiation [Fig. 6C; RM anova with drug (chelerythrine vs. control) as a between-subjects factor and time (0–5 min and 45–50 min after pTBS) as a within-subject factor, main effect of drug, *F*_1,15_ = 4.9, *P*<0.05, no further effects or interactions, all *F*<3.4, *P*>0.09]. Hence, both GluA1-independent LTP and the later phase of conventional LTP in wild-type mice require PKC, possibly the PKMζ isoform.

## Discussion

### The role of GluA1 in LTP

It is well established that the GluA1 subunit is important for the expression of LTP. Phosphorylation of GluA1 has been shown to increase the conductance of AMPAR (Benke *et al.*, 1998; Derkach *et al.*, 1999), and the interaction of trafficking proteins and kinases with GluA1 regulates the activity-dependent surface expression of AMPAR (Hayashi *et al.*, 2000; Lu *et al.*, 2001; Shi *et al.*, 2001; Piccini & Malinow, 2002). Furthermore, Zamanillo *et al.* (1999) previously reported that *Gria1*^−/−^ mice did not express CA3–CA1 LTP. However, this view has been challenged by Hoffman *et al.* (2002), who provided initial evidence that a GluA1-independent form of potentiation can be expressed in these animals, when an intracellular, paired theta-burst induction protocol is used. Whereas the induction paradigm used by Hoffman *et al.* (2002) not only potentiated EPSPs in the paired pathway but also the unpaired control pathway, and the resulting, GluA1-independent potentiation developed gradually over 30 min, we have demonstrated here that extracellular pTBS can induce robust, input-specific, GluA1-independent LTP that is rapidly established within 5–10 min. However, GluA1-independent LTP could not be induced with a single weak tetanus (also see Zamanillo *et al.*, 1999; Jensen *et al.*, 2003), and lacks the initial, rapidly decaying potentiation seen in wild-type mice. Thus, synaptic potentiation at CA3–CA1 synapses appears to comprise a rapidly decaying, GluA1-dependent potentiation, and a more persistent, GluA1-independent potentiation that requires a stronger induction paradigm. Our initial findings raise the question about the extent to which induction and expression mechanisms of these two forms of potentiation are distinct or shared. We have established GluN2B-containing NMDAR, CaM kinase, PKC and nNOS as part of the signalling cascade that mediates GluA1-independent LTP. Of those, only CaM kinase was also required for the induction of the early, rapidly decaying, probably GluA1-dependent form of potentiation in wild-type mice. Although our findings are not sufficient to explain how GluA1-independent LTP is finally expressed, CaMKII, nNOS and PKC are known to regulate the activity-dependent, synaptic expression of AMPAR by interacting with the GluA2 subunit (Daw *et al.*, 2000; Gardner *et al.*, 2005; Oh & Derkach, 2005; Lin & Huganir, 2007; Yao *et al.*, 2008).

### GluA1-independent LTP requires GluN2B-containing NMDAR

Both GluA1-independent LTP and LTP in wild-type mice are NMDAR dependent. Unlike LTP in wild-type mice, GluA1-independent LTP was strongly attenuated by the GluN2B subunit-selective NMDAR antagonist Ro 25-6981. Because GluN2B-containing NMDAR are preferentially, although not exclusively, located extrasynaptically (Stocca & Vicini, 1998; Tovar & Westbrook, 1999; Steigerwald *et al.*, 2000), and a single weak tetanus failed to induce LTP in *Gria1*^−/−^ mice, the induction of GluA1-independent LTP might require a more global Ca^2+^ signal. Consistent with this possibility, Hoffman *et al.* (2002) found that the early, possibly GluA1-dependent phase of potentiation and the later, possibly GluA1-independent phase of LTP in wild-type mice are differentially sensitive to internal Ca^2+^ buffers. Alternatively, or additionally, the relative synaptic GluN2B/GluN2A subunit composition might be different in the *Gria1*^−/−^ mice, as has recently been described in *Kv4.2*^−/−^ mice (Jung *et al.*, 2008).

Although we show an important role of GluN2B-containing NMDAR in the induction of GluA1-independent LTP, the contribution of GluN2A-containing NMDAR remains uncertain. NVP-AAM077 (50 nm) had no effect on LTP in *Gria1*^−/−^ mice, but significantly reduced the early potentiation in wild-type mice, suggesting a more important role of GluN2A in this early, possibly GluA1-dependent potentiation. At the higher concentration used (400 nm), NVP-AAM077 blocked LTP in both genotypes, but does not distinguish between GluN2A- and GluN2B-containing receptors (Berberich *et al.*, 2005; Neyton & Paoletti, 2006).

### Downstream signalling in GluA1-independent LTP: CaM kinase, PKC and nNOS

The CaM kinase inhibitor KN-62 blocked GluA1-independent LTP and both the early and the later phases of LTP in wild-type mice. The necessity of CaMKII for at least the early phases of conventional LTP is well established (see Lisman *et al.*, 2002), but one of the main CaMKII target proteins implicated in this process is the GluA1 subunit (Barria *et al.*, 1997; Benke *et al.*, 1998; Lee *et al.*, 2000, 2003). Our findings suggest that CaM kinase target proteins other than GluA1 are involved in the induction and/or expression of sustained GluA1-independent LTP. Interestingly, an interaction of CaMKII with the GluN2B NMDAR subunit has been reported to be necessary for pairing-induced LTP in wild-type mice (Barria & Malinow, 2005). However, because KN-62 inhibits other CaM kinases in addition to CaMKII (Enslen *et al.*, 1994), we cannot attribute the effect of KN-62 to inhibition of CaMKII (Wayman *et al.*, 2008), and it is possible that the early phase of LTP and the GluA1-independent form of LTP are mediated by distinct members of the CaM kinase family.

We also showed that GluA1-independent LTP is attenuated by chelerythrine, a potent, cell-permeable PKC inhibitor that binds to the catalytic domain of PKC. Postsynaptic PKC is activated following strong NMDAR activation and release of Ca^2+^ from intracellular stores, experimentally achieved by stronger, repetitive LTP induction paradigms (Bliss & Collingridge, 1993; Raymond, 2007). Activated PKC has been shown to regulate synaptic AMPAR trafficking by phosphorylation of GluA1 (Boehm *et al.*, 2006) and AMPAR surface expression by phosphorylation of GluA2 (Daw *et al.*, 2000; Gardner *et al.*, 2005; Lin & Huganir, 2007) and/or PICK1, a GluA2-linked trafficking protein (Staudinger *et al.*, 1995, 1997; Perez *et al.*, 2001). Furthermore, the PKC isoform PKMζ increases synaptic AMPAR and excitatory synaptic transmission (Ling *et al.*, 2006), probably by an NSF/GluA2-dependent mechanism that was recently found to maintain late-phase LTP through persistent trafficking of AMPAR to the synapse (Yao *et al.*, 2008). Thus, PKC-regulated synaptic trafficking of GluA2 might contribute to GluA1-independent LTP.

Finally, we showed that GluA1-independent LTP is attenuated by the nNOS inhibitors nNOSi I and V-LNIO. Because the exact intracellular concentrations of these drugs under our experimental conditions are unknown, we cannot rule out the possibility that the inhibitors also affected other NOS isoforms. Indeed, it was recently reported that endothelial NOS (eNOS) is also involved in a GluA1-independent form of LTP (Phillips *et al.,* 2008). In wild-type mice, nNOS is known to produce nitric oxide upon NMDAR activation (Bredt & Snyder, 1989; Garthwaite *et al.*, 1989; Brenman *et al.*, 1996; Brenman & Bredt, 1997), and nitric oxide can S-nitrosylate NSF (Huang *et al.*, 2005), another protein implicated in GluA2 trafficking. S-nitrosylation of NSF results in an increased surface expression of GluA2-containing AMPAR in cultured cerebellar and hippocampal neurons (Huang *et al.*, 2005; Sossa *et al.*, 2006, 2007). Indeed, Kakegawa & Yuzaki (2005) showed that such a mechanism could underlie GluA1-independent LTP at cerebellar parallel fibre–Purkinje cell synapses. Although nNOS inhibitor I in our hands did not affect LTP in wild-type mice, a previous study reported that nNOS inhibitors produce a major loss of particularly the later phases of LTP (Hopper & Garthwaite, 2006). Taken together with the evidence that GluA1-independent LTP might also require PKMζ, it is possible that GluA1-independent mechanisms mediate or maintain later phases of LTP in wild-type mice.

### Reduced synaptic transmission in Gria1^−/−^ mice

Field excitatory synaptic transmission in *Gria1*^−/−^ mice was reduced relative to wild-type mice. Given the evidence that AMPAR in CA1 pyramidal neurons of wild-type mice mainly consist of GluA1/GluA2 heteromers, fewer GluA2/GluA3 complexes and some GluA1 homomers (Wenthold *et al.*, 1996), the observed reduction in excitatory synaptic transmission in *Gria1*^−/−^ mice is not surprising. On the other hand, the majority of AMPAR in CA1 pyramidal neurons are found at extrasynaptic sites (Shi *et al.*, 1999), so GluA1-lacking AMPAR might be able to compensate for the missing GluA1-containing AMPAR at the synapse. Indeed, Zamanillo *et al.* (1999) reported that AMPAR currents in *Gria1*^−/−^ mice are strongly reduced at the soma, but largely unaltered at synaptic sites on the proximal dendrites, which seems to be at odds with our findings. While we cannot explain this apparent discrepancy, a more recent study showed that AMPAR-mediated currents at more distal dendritic sites in *Gria1*^−/−^ mice are prominently reduced (Andrasfalvy *et al.*, 2003), and Jensen *et al.* (2003) reported a strongly reduced synaptic AMPA/NMDA current ratio in adult (>P42) *Gria1*^−/−^ mice.

### Does GluA1-independent LTP exist in wild-type mice?

A common problem of working with genetically modified mice is that deletion of a gene might alter developmental processes. Cellular, transcriptional and/or nuclear plasticity might compensate for the lack of a gene by recruiting mechanisms/proteins/genes not normally used for a function, or by developing entirely new mechanisms. Thus, the GluA1-independent, GluN2B-, nNOS- and PKC-dependent form of LTP that we describe in *Gria1*^−/−^ mice might be the result of altered development or the recruitment of GluA1-independent mechanisms not present in wild-type mice. However, similar cellular signalling cascades involving nitric oxide and PKC have previously been shown to mediate AMPAR exocytosis in cultured hippocampal neurons (Sossa *et al.*, 2007) and mediate activity-regulated GluA2 surface expression in wild-type animals (Daw *et al.*, 2000; Hayashi *et al.*, 2000; Passafaro *et al.*, 2001; Yao *et al.*, 2008). Whether GluA1-dependent and GluA1-independent mechanisms in wild-type mice are really as separable as our data from *Gria1*^−/−^ mice suggest remains to be investigated. Because most AMPAR at hippocampal synapses of wild-type mice consist of either GluA1/GluA2 or GluA2/GluA3 heteromers (Wenthold *et al.*, 1996), an interaction of both GluA1- and GluA2-linked (and possibly GluA3-linked) trafficking/anchoring cascades is likely to determine the synaptic expression and maintenance of these receptors, and therefore the ultimate magnitude and persistence of LTP. Hence, GluA1-independent LTP as uncovered in *Gria1*^−/−^ mice might be redundant for some but not all phases of LTP in wild-type mice, which would explain why the first hour of LTP in wild-type mice neither required GluN2B nor nNOS.

Interestingly, the dissociation of GluA1-dependent and GluA1-independent potentiation is matched by a dissociation of two types of hippocampus-dependent spatial memory: the GluA1 subunit is necessary for spatial working memory, whereas the gradual acquisition of spatial reference memory tasks is GluA1-independent (Reisel *et al.*, 2002; Schmitt *et al.*, 2003; Sanderson *et al.*, 2007). In neither case (plasticity or behaviour) is the short-term process required for the long-term process to manifest. This highlights the importance of distinguishing between different plasticity mechanisms when addressing the relationship between synaptic plasticity and memory.

## Abbreviations

aCSF: artificial cerebrospinal fluid
AMPA: α-amino-3-hydroxy-5-methyl-4-isoxazole proprionic acid
AMPAR: AMPA-type glutamate receptor(s)
CaMKII: Ca^2+^/calmodulin-dependent protein kinase II
CPP: 3-(R-2-carboxypiperazin-4-yl)-propyl-1-phosphonic acid
EPSP: excitatory postsynaptic potential
fEPSP: field excitatory postsynaptic potential
KN-62: 4-[(2*S*)-2-[(5-isoquinolinylsulphonyl)methylamino]-3-oxo-3-(4-phenyl-1-piperazinyl)propyl] phenylisoquinolinesulphonic acid ester
LTP: long-term potentiation
NMDA: *N*-methyl-d-aspartate
NMDAR: NMDA-type glutamate receptor(s)
nNOS: neuronal nitric oxide synthase
NSF: *N*-ethylmaleimide-sensitive factor
NVP-AAM077: (*R*)-[(*S*)-1-(4-bromo-phenyl)-ethylamino]-(2,3-dioxo-1,2,3,4-tetrahydroquinoxalin-5-yl)-methyl-phosphonic acid
PICK1: protein interacting with C kinase-1
PKC: protein kinase C
PKMζ: protein kinase Mζ
PPR: paired-pulse ratio
pTBS: paired synaptic-antidromic theta-burst stimulation
RM: repeated-measures
Ro 25-6981: (*αR,βS*)-α-(5-hydroxyphenyl)-β-methyl-4-(phenylmethyl)-1-piperidinepropanol
